# Cognitive Phenotypes, Brain Morphometry and the Detection of Cognitive Decline in Preclinical AD

**DOI:** 10.3233/BEN-2009-0229

**Published:** 2009-10-21

**Authors:** Mark W. Jacobson, Linda K. McEvoy, Anders Dale, Christine Fennema-Notestine

**Affiliations:** ^1^Veterans Affairs San Diego Healthcare SystemSan DiegoCAUSA; ^2^Department of PsychiatryUniversity of California San DiegoSan DiegoCAUSA; ^3^Department of NeurosciencesUniversity of California San DiegoSan DiegoCAUSA; ^4^Department of RadiologyUniversity of California San DiegoSan DiegoCAUSA

**Keywords:** MRI, Alzheimer’s disease, cognition, morphometry, asymmetry

## Abstract

Identifying a preclinical phase of Alzheimer’s Disease (PCAD) that is distinct from cognitive changes in healthy aging continues to be a major research focus. Combining neuropsychological and neuroimaging methodologies should improve our ability to differentiate healthy from pathological aging, although studies that utilize both methods often result in equivocal findings, possibly due to variability in cognitive test performance that may be capturing distinct phenotypes. One method of capturing this cognitive variability is to utilize contrasting neuropsychological tests to identify subgroups representative of distinct cognitive phenotypes, and determine whether differences in brain morphometry support these classifications. We review several approaches to defining cognitive subgroups, and we consider the possibility that cognitive asymmetry might provide one means of identifying both functional and structural changes associated with aging and dementia.

## 1. Introduction

Distinguishing healthy cognitive aging from subtle changes characteristic of a preclinical phase of Alzheimer's Disease (PCAD) has become increasingly important as new pharmacologic interventions become available, and as our population ages. Increased longevity is accompanied by a concurrent rise in Alzheimer's disease (AD), with estimated incidence increasing from 0.08% per year at age 65 to 6.48% per year at age 85 [23]. Improving our ability to identify AD at the earliest stages before significant neurodegenerative changes occur could have a tremendous impact on potential neuroprotective therapies, early interventions with individuals at increased risk, and long-term planning issues. Neuropsychological tests have been one of the mainstays of early detection, with some studies suggesting that subtle changes in cognition can be detected ten years or more prior to functional changes and a subsequent clinical diagnosis of AD [9]. However, distinguishing age-related deficits from preclinical AD can be problematic given the increased heterogeneity of cognitive profiles in older adults. This increased variability in test performance probably reflects a cognitive continuum that ranges from stable, preserved abilities and benign age-related changes, to subtle deficits more suggestive of the early stages of a dementing process. One method of capturing this cognitive variability is to identify elderly subgroups representative of distinct cognitive phenotypes, and determine whether differences in brain morphometry support these classifications.

Because cognitive profiles of PCAD share many common elements with healthy aging, investigators

ISSN 0953-4180/09/$17.00 © 2009 – IOS Press and the authors. All rights reserved have suggested that a combination of neuropsychological testing and neuroimaging is likely to be the best method to distinguish the two [8]. Both techniques can be sensitive indicators of PCAD despite disagreement about whether morphometric changes are typically detected prior to subtle cognitive changes in PCAD [33, 43]. Many studies clearly find age-related changes in brain structures predictive of neuropsychological functioning [3], but there has not always been consistent, robust findings that link brain structure with function [45, 49]. In terms of neuroimaging, equivocal findings have been partially attributed to differences in methodologies [47], and a paucity of large-scale studies with sufficient sample sizes, or prospective longitudinal designs [21,22] (see Fennema-Notestine, McEvoy et al. in this issue). However, the current discussion will focus on characteristics of cognitive testing, and methods of analysis that might contribute to ambiguous findings in brain/behavior associations. We will consider whether methods that capture cognitive heterogeneity by identifying distinct cognitive subgroups might contribute to improved discrimination between healthy and pathological aging.

Many cognitive studies of early detection have focused on characteristics of a unitary process or a modal pattern of evolving deficits in elderly at increased risk for AD [28]. There is considerable evidence that the most typical progression in AD begins with learning and memory changes, progressing to more widespread deficits in semantic networks, language skills, praxis and executive functions, eventually affecting most cognitive skills. However, many of the cognitive profiles that typify both MCI and AD also reveal considerable heterogeneity [5,32], and suggest that this variability in cognitive abilities is a feature of PCAD [52]. The probable number and complexity of PCAD profiles increases when we consider that the cognitive changes pathognomic for AD could be superimposed on an evolving pattern of age-related changes in other cognitive domains [4,14,38]. That is, amnestic changes or deficits in semantic networks common to prodromal stages of AD could occur in conjunction with age-related changes in processing speed, cognitive control, and encoding efficiency [48,50]. Given the complexity of these processes, developing cognitive profiles that reflect heterogeneous changes might improve our ability to understand the corresponding changes in neural networks.

The presence of cognitive subgroups that reflect multiple, modular cognitive deficits has been a longstanding consideration in AD diagnosis and nosology [26, 30,59], helping to identify patterns of neuropathological changes [1]. For example, recent studies identified four AD subgroups based on patterns of atrophy [41]; two AD subgroups based on distribution of neurofibrillary tangles [31], and the presence of comorbid factors affecting cognition contributed to six clearly defined AD subgroups [25]. Confirmation of distinct AD subgroups identified by patterns in amyloid-B and neurofibrillary degeneration could even benefit development of subtype-specific therapeutic drugs [16]. Identifying multiple, qualitatively distinct subgroups within the larger aging population may serve to better characterize the variability of cognitive decline in AD [29], and possibly improve our ability to predict such decline on the basis of brain morphometry.

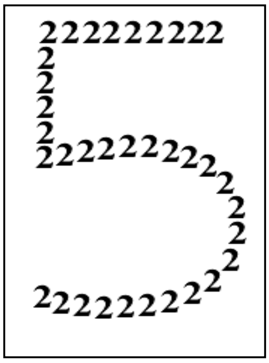

Fig. 1. Navon-type stimuli from attention-shifting task: Global "5" with local "2" target.

One method of identifying potential subgroups in elderly cohorts with increased risk for AD has been to define asymmetric cognitive profiles that are based on dissociations between two abilities or domains [18–20]. One distinct advantage to this approach, particularly for cross-sectional research, is that it facilitates interpretation of intra-individual cognitive patterns by comparing consistency of performance across multiple tests. There is an additional advantage, in that analyses of a single domain using measures of central tendency could limit detection of potential subgroups with disparate abilities [30]. For example, contrasting recall abilities using two different modalities resulted in detection of two distinct subgroups in the early stages of AD; one with relatively preserved verbal memory but impaired spatial memory, and a second group with the opposing pattern [6]. A number of studies have detected subgroups with asymmetric cognitive profiles in early AD [46] a trend that could reflect asymmetric neurodegeneration early in the course of the disease [44]. Interestingly, some studies of brain morphometry in older cohorts suggest that a reduction of expected structural asymmetry in hippocampal volume

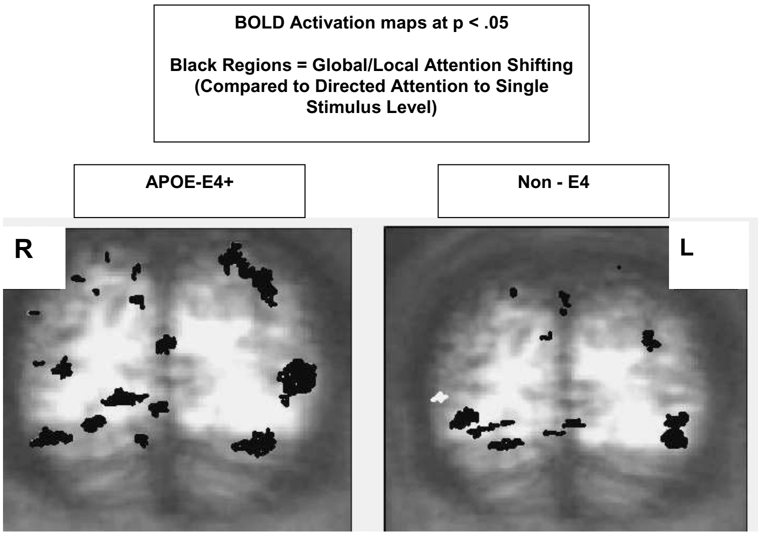

Fig. 2. Coronal views of BOLD activation with T1-weighted MRI in APO$\varepsilon$4+ and non-$\varepsilon$4 groups.

may be associated with both APOE-$\varepsilon$4 genotype, and a greater likelihood of progression to AD [13,54] while others find that asymmetry is not influenced by age or disease state [56].

Given these findings, asymmetric cognitive abilities in these subgroups may indicate an increased risk for progression to AD. A retrospective analysis noted that large cognitive discrepancies (greater than one standard deviation) between verbal naming and visuoconstruction performance in healthy older adults were associated with a subsequent change in diagnosis to "at-risk" or "possible AD" within two years of assessment [18]. In a follow-up study, older adults with APOE- $\varepsilon$4+ genetic risk were compared with non-$\varepsilon$4 subjects on tests of auditory and visual attention span [19]. Despite comparable performance on the tests, the $\varepsilon$4+ subgroup had larger verbal/spatial discrepancies relative to the non-$\varepsilon$4 subgroup. A similar pattern of larger cognitive discrepancies in APOE-$\varepsilon$4+ elderly was noted on the switching condition of a Stroop interference/inhibition task (D-KEFS Color-Word Interference subtest) [15].

Distinct subgroups of healthy APOE $\varepsilon$4+ elderly also showed asymmetric recall on a task incorporating Navon-type stimuli (Fig. 1) that is sensitive to lateralized hemispheric processing and unilateral brain damage [10]. (Large, global gestalts are preferentially processed by right-hemisphere brain regions, while small, detailed local stimuli are left-hemisphere dependent.) Despite comparable overall recall, the $\varepsilon$4+ group contained a subgroup with disproportionate recall of global relative to local details, and a second subgroup with preferential local recall [20]. It is possible that this difficulty shifting between stimulus components in the $\varepsilon$4+ subgroup could be a precursor of the attention-shifting deficits found in early AD [37]. To extend these findings, an FMRI task was utilized to determine if the neural substrates underlying visual attention-shifting also would reflect the presence of genotype subgroups [57]. In this study, participants identified a series of target numbers that shifted between global and local stimulus levels at pseudo-random intervals (see Fig. 1). A preliminary comparison of BOLD activation in 14 genotyped adults over age 65 (seven $\varepsilon$ 3/4 versus seven $\varepsilon$ 2/3 or 3/3) showed a greater spatial extent of blood oxygenation level-dependent changes (BOLD) in the $\varepsilon$4+ group, primarily in left frontal and bilateral parietal brain regions relative to a matched non-$\varepsilon$4 group. Figure 2 shows significant task-related BOLD changes in both groups (overlaid on group composites of T1 weighted structural MRIs) that contrast activation in the attention shifting condition (black areas), and directed attention to a single perceptual level (white areas) based on AFNI single group $t$-tests with voxel clusters $>$ 300 mm$^3$ at $p < 0.05$. Although these preliminary data might suggest APOE-related differences in brain activation, the BOLD changes also occurred in brain regions that are among the most common sites of general, age-related structural changes and compensatory brain activity [14] regardless of genotype.

Larger, longitudinal studies of aging are needed to determine if APOE-$\varepsilon$4 subgroups demonstrating asymmetric cognitive profiles represent a preclinical phase of AD, or whether they indicate longstanding cognitive phenotypes that become more apparent with ag-

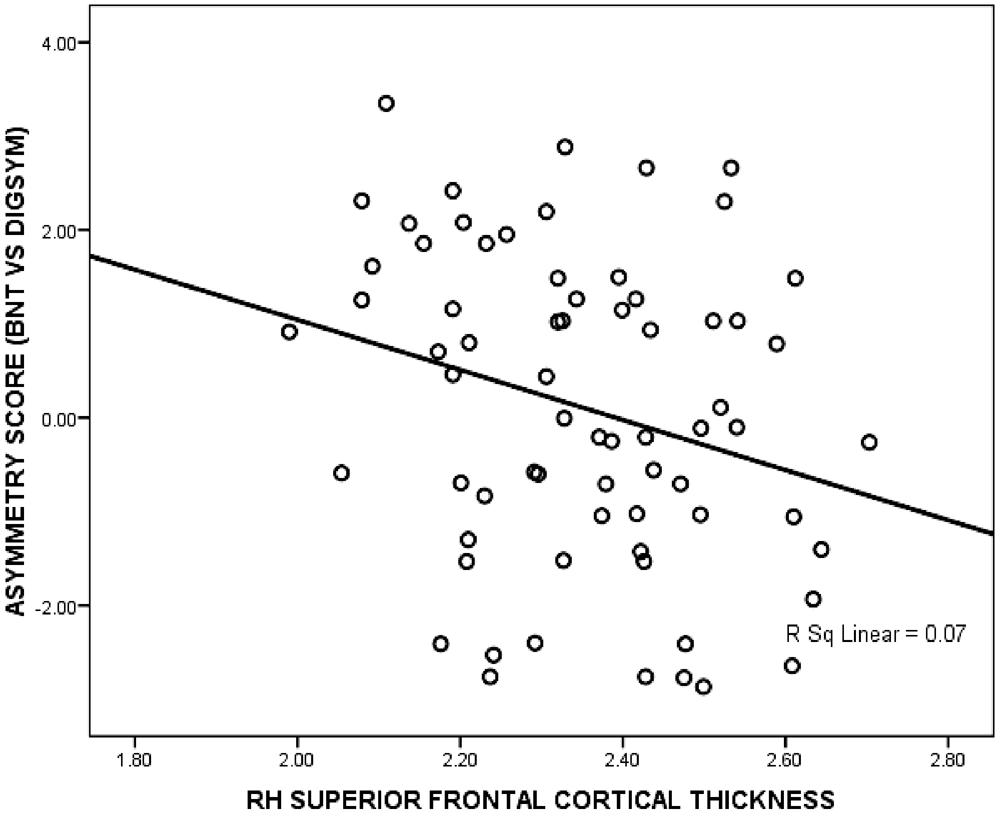

Fig. 3. Boston naming/digit symbol asymmetry and right frontal cortical thickness.

ing [40]. The examples that follow are based on data publicly available from the multi-site Alzheimer's Disease Neuroimaging Initiative (ADNI), an important effort designed to facilitate the scientific evaluation of neuroimaging and other biomarkers in the onset and progression of MCI and AD [17,34,35]. The large sample of healthy older controls and individuals with MCI collected as part of the ADNI (see www.adni-info.org) could provide additional evidence regarding the frequency of $\varepsilon4+$ subgroups with cognitive asymmetry, and whether these profiles have structural brain correlates. From data collected at baseline, an asymmetry index was used to identify performance discrepancies between Boston Naming Test, [BNT] and Digit Symbol Test, [DSYM] in a subset of older healthy controls from the ADNI cohort ($n$ = 192) [58]. As with previous studies, the higher risk, $\varepsilon4+$ subgroup had a significantly higher proportion of individuals with a large z-score difference (greater than 1.5 SD) relative to those without the $\varepsilon4$ genetic risk ($\chi^2$ = 6.2, $p$ = 0.01). In addition, cognitive asymmetry predicted cortical thickness in right superior frontal (F = 3.24, $p$ = 0.04 see Fig. 3) and medial orbitofrontal cortex (F = 4.12, $p$ = 0.02) with increased asymmetry associated with thinner cortex after controlling for gender and estimated IQ. In addition, asymmetry scores predicted subcortical volume of thalamus (F = 7.14, $p < 0.01$) and caudate (F = 6.32, $p < 0.01$) (controlling for intracranial volume and gender). The asymmetry direction interacted with morphometry measures: cognitive asymmetry resulting from lower DSYM scores relative to BNT was associated with greater reduction in bilateral caudate volume compared to an asymmetry score caused by lower relative BNT scores (Fig. 4). Examining brain morphometry measures associated with the BNT and DSYM scores individually resulted in consistently weaker (or non-significant) associations. This suggests that computation of indices to characterize cognitive subgroups could highlight subtle group differences in brain morphometry that were not associated with individual cognitive measures. Longitudinal studies will determine if such indices could identify groups

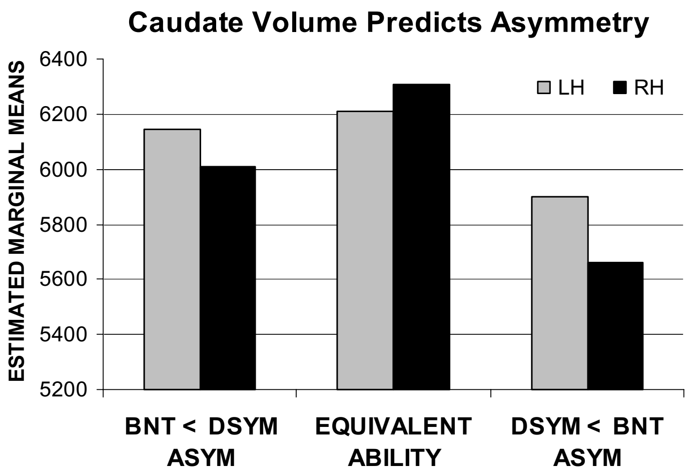

Fig. 4. Bar graph of caudate volumes in healthy elderly subgroups with more than 1.5 SD cognitive asymmetry.

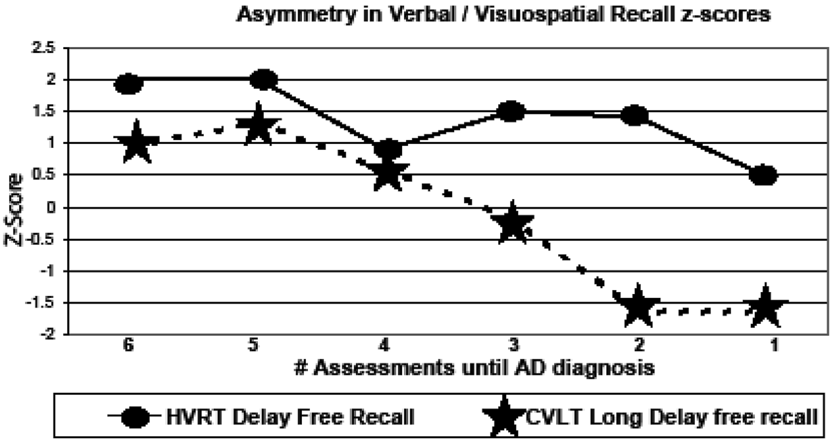

Fig. 5. Six longitudinal assessments of verbal/spatial asymmetry in preclinical AD case study.

with progressive structural changes and increased risk for cognitive decline or AD.

In addition to the use of test contrasts for identifying asymmetric subgroups with differing risk of decline, comparing performance on two disparate measures yields an additional benefit by revealing possible intra-individual changes in cognition. This method of interpreting probable decline using cross-sectional data has been used to detect the presence of neurodegenerative changes in a variety of patient groups. A priori test selection is based on contrasts between a cognitive domain that is resilient to injury or a neurodegenerative process, and a second domain that is more sensitive to impairment [53]. Alternatively, a contrast between two domains could be established to mirror a specific neurodegenerative process as well (e.g. lateralized atrophy in medial temporal regions). This type of contrast often reveals a probable decline in functioning in individuals with high premorbid abilities whose performance has not yet reached a level of true impairment. Figure 5 displays an illustrative example, obtained from a six-year, longitudinal case study of an 80-year old female healthy control participant who was diagnosed with possible AD following the final assessment. The graph depicts norm-referenced $z$-scores on the delayed recall conditions of a verbal learning test. (California Verbal Learning Test) and a test of recall for designs (Heaton Visual Reproduction Test). Although the participant's memory performance would not be considered significantly impaired until the final assessment, performance discrepancies greater than one standard deviation revealed increasing asymmetries between verbal and spatial modalities at a time when her overall cognition was not significantly impaired (i.e.

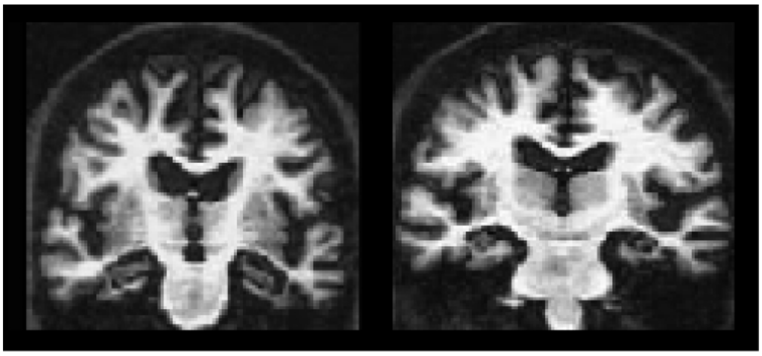

Fig. 6. MRI illustrating atrophic changes 2 years prior to AD diagnosis.

Mattis Dementia Rating Scale total score > 138 points). The cognitive discrepancies were apparent in multiple cognitive domains, and may have signaled a decline in cognitive abilities even though her age-adjusted performance on the individual tasks had not yet reached the impaired range. Age-adjusted norms showed that she maintained an average to above average performance in most domains until the year prior to her change in diagnosis, even though structural neuroimaging after the fifth assessment confirmed significant generalized cortical atrophy most prominent in medial temporal, and hippocampal regions (see Fig. 6).

This individual's cognitive profile was characteristic of a subgroup of patients who present with a precipitous rate of decline [42] rather than a gradual onset of impairment. However, the interpretation of "impairment" for test performances in the "oldest-old" subgroup depends greatly on the normative comparison group – an additional factor that may contribute to equivocal findings in structural/functional brain relationships. Comparing cognitive tasks, or creating composite domain scores will often require transformation of raw scores into standardized scores to better facilitate inter-test comparisons. In certain tasks, such as those requiring processing speed, even minor age-related changes can have a considerable impact on score variability, and consequently, on the interpretation of "impairment" at the individual level. For example, an examination of ADNI participants over age 65 suggested that performance on Trails B would vary within age-specific subgroups in this cohort (see Fig. 7 showing mean and standard error for Trails B in four older age subgroups).

Scores that might be indicative of healthy cognitive aging in an 85-year old could be considered in a deficit range in a younger subgroup. Because the strength of relationships between cognitive abilities and morphometry measures can vary by age groups [2,55], failing

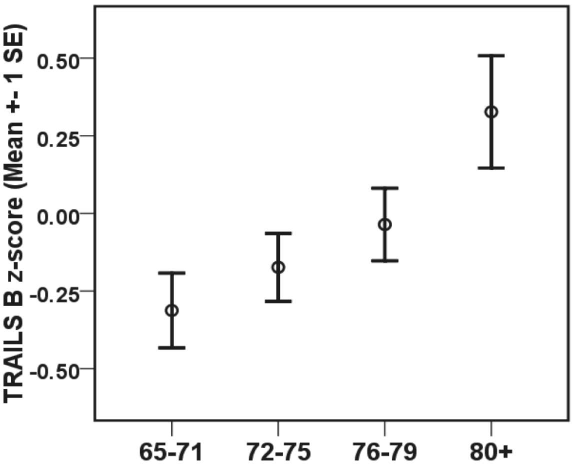

Fig. 7. Trails B performance in four subgroups of healthy older adults: Mean and standard error.

to stratify performance in the "oldest" old subgroups could partially account for variable findings relating brain structure to function in this group.

To continue investigating age subgroup effects, we examined the healthy older control group from the ADNI cohort (NC), dividing them into three similar sized subgroups, age 63–72 ($n = 65$), age 73–79 ($n = 63$), and age 80+ ($n = 69$). We also converted Trails A and Trails B scores to z-scores based on the entire NC sample. We used this z-score difference (Trails B minus Trails A [TrBz-TrAz]) to isolate visuospatial sequencing ability while partially controlling for processing speed. We then computed a cortical thickness measure [56] for superior frontal cortex (SFC) regions controlling for gender. We found trends for interactions between age subgroups and both TrBz-TrAz scores ($F$ (2,193) $= 3.9$ $p < 0.021$) and right hemisphere superior frontal thickness ($F$ (2,193) $= 3.01$ $p = 0.051$) with the oldest-old subgroup significantly different from the youngest-old subgroup. Linear multiple

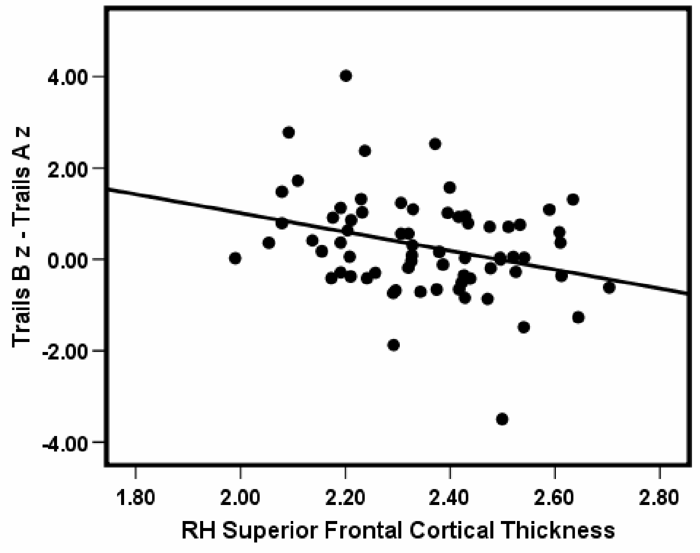

Fig. 8. Scatterplot of trails B z-score minus trails A z-score and RH superior frontal cortical thickness.

regressions showed that only the oldest-old subgroup's performance on TrBz-TrAz was a significant predictor of right hemisphere SFC after entering gender in the model (Adj. R square $= 0.202$; F change $= 4.65$ $p = 0.035$, standardized beta $= -0.240$). TrBz-TrAz was not a significant predictor of cortical thickness in either of the two "younger" old groups (both F change $< 1.0$; $p > 0.10$). Those in the oldest-old subgroup with faster visuospatial sequencing had thicker cortex in our region of interest (see Fig. 8.) Accounting for subtle differences in performance in older age subgroups could strengthen relationships between cognitive performance and brain morphometry.

Characterizing cognitive phenotypes that might be associated with increased risk for AD will require methods that account for multiple sources of variability in cognitive test scores. These include a consideration of potential ceiling and floor effects in cognitive measures [39], and the necessity of matching test variables on levels of difficulty [24]. There are also many demographic elements that can interact with age and cognitive performance such as premorbid IQ, socioeconomic status, gender, and educational level [7,12, 36] increasing variability in neuropsychological performance. New brain morphometry methodologies that yield increased regional specificity [11,27,51,56] will require new methods of fractionating and merging components of cognition in order to improve our ability to predict cognitive abilities based on brain structures in healthy aging and PCAD. Developing cognitive indices that capture cognitive heterogeneity through asymmetric, or stratified, age subgroups may uncover stronger relationships with morphometry measures.

## Acknowledgements

This work was supported by grants from the Department of Veterans Affairs (Advanced Career Dev. Award – MWJ), the National Center for Research Resources (U24 RR021382 – CFN, AMD; DJH); National Institute on Aging (R01AG22381 – CFN, LKM, AMD; K01AG029218 – LKM); and. This review refers to important work facilitated by the Alzheimer's Disease Neuroimaging Initiative (ADNI; Principal Investigator: Michael Weiner; NIH grant U01 AG024904), which funded data collection and sharing. ADNI is funded by the National Institute on Aging, the National Institute of Biomedical Imaging and Bioengineering (NIBIB), and through generous contributions from the following: Pfizer Inc., Wyeth Research, Bristol-Myers Squibb, Eli Lilly and Company, GlaxoSmithKline, Merck & Co. Inc., AstraZeneca AB, Novartis Pharmaceuticals Corporation, Alzheimer's Association, Eisai Global Clinical Development, Elan Corporation plc, Forest Laboratories, and the Institute for the Study of Aging, with participation from the U.S. Food and Drug Administration. Industry partnerships are coordinated through the Foundation for the National Institutes of Health. The grantee organization is the Northern California Institute for Research and Education, and the study is coordinated by the Alzheimer's Disease Cooperative Study at the University of California, San Diego. ADNI data are disseminated by the Laboratory of Neuro Imaging at the University of California, Los Angeles.
